# Relaparotomy pseudoaneurysm repair after distal pancreatectomy with celiac axis resection (DP-CAR): a case report

**DOI:** 10.1093/jscr/rjae204

**Published:** 2024-04-04

**Authors:** Shun Nakamura, Kazuhiro Tada, Junya Mita, Kengo Fukuzawa

**Affiliations:** Department of Surgery, Oita Red Cross Hospital, 3-2-37 Chiyomachi, Oita-shi, Oita 870-0033, Japan; Department of Surgery, Oita Red Cross Hospital, 3-2-37 Chiyomachi, Oita-shi, Oita 870-0033, Japan; Department of Surgery, Oita Red Cross Hospital, 3-2-37 Chiyomachi, Oita-shi, Oita 870-0033, Japan; Department of Surgery, Oita Red Cross Hospital, 3-2-37 Chiyomachi, Oita-shi, Oita 870-0033, Japan

**Keywords:** distal pancreatectomy with celiac axis resection, pseudoaneurysm, hemostasis

## Abstract

A 76-year-old man underwent distal pancreatectomy with celiac axis resection (DP-CAR) after preoperative chemotherapy for pancreatic cancer with celiac artery invasion. Although postoperative pancreatic leakage and ischemia-induced bile fistula developed, the patient’s condition remained stable with good drainage. On postoperative Day 47, a pseudoaneurysm developed at the junction of the gastroduodenal artery and proper hepatic artery. However, cannulation of the guidewire was difficult, and relaparotomy pseudoaneurysm repair was performed. On postoperative Day 56, a pseudoaneurysm reappeared at the same site, and relaparotomy was performed again. On postoperative Day 61, CT confirmed the disappearance of the pseudoaneurysm and preservation of the right and left hepatic arteries. The patient was discharged 107 days postoperatively. Interventional radiology (IVR) remains the best technique to achieve hemostasis for pseudoaneurysms. However, this case demonstrates that even when hemostasis by IVR is difficult, relaparotomy pseudoaneurysm repair after DP-CAR may be useful after some postoperative.

## Introduction

Distal pancreatectomy with celiac axis resection (DP-CAR) has been considered useful for locally advanced pancreatic body regions with celiac artery and common hepatic artery (CHA) invasion; however, its indications are limited by high complication rates [1].

## Case report

**Figure 1:**
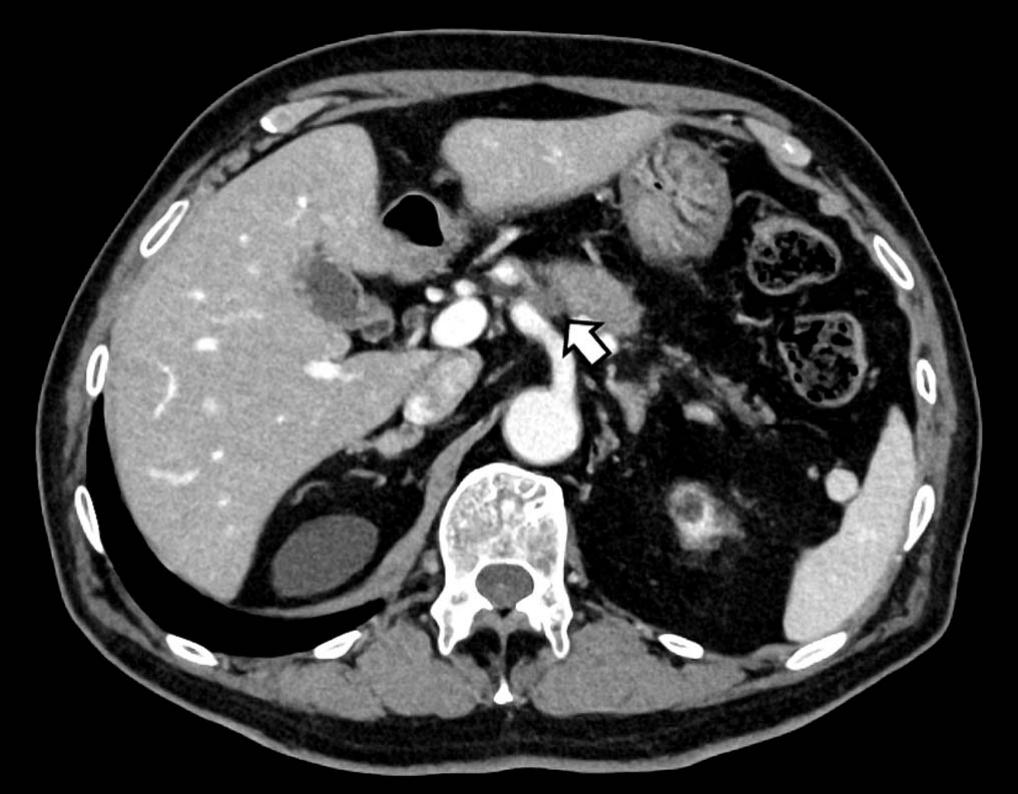
Computed tomography after preoperative chemotherapy showing tumor invasion on the left side of the celiac artery.

**Figure 2:**
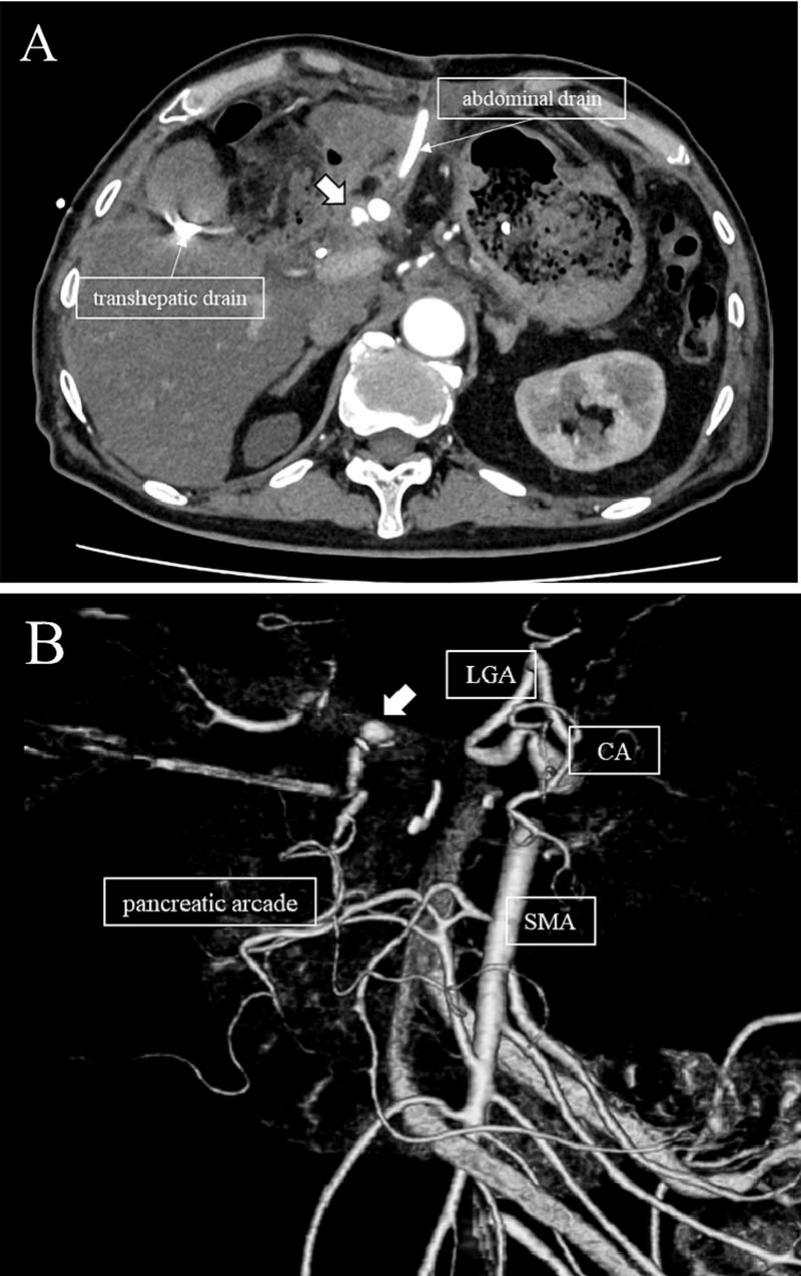
(A, B) CT on postoperative day 48. Contrast-enhanced CT showing a pseudoaneurysm at junction of the GDA and PHA (arrow).

A 76-year-old man underwent DP-CAR after eight courses of gemcitabine and nab-paclitaxel therapy as preoperative chemotherapy for pancreatic body cancer with celiac artery invasion (Fig. 1). As the celiac axis had not invaded into the bifurcation of the left gastric artery, a modified DP-CAR (left gastric-preserving) was performed. Although post operative pancreatic fistula (POPF) developed on postoperative Day (POD) 5, and biliary fistula developed on POD 17. Bile leak was thought to be caused by the rupture of the choledochal duct due to bile duct ischemia caused by CHA transection. Subsequently, both the POPF and biliary fistulae were drained and fistulated. However, on POD 47, the drainage fluid became bloody. Contrast-enhanced computed tomography (CeCT) revealed a pseudoaneurysm at the junction of the gastroduodenal artery (GDA) and proper hepatic artery (PHA) (Fig. 2A and B).

**Figure 3:**
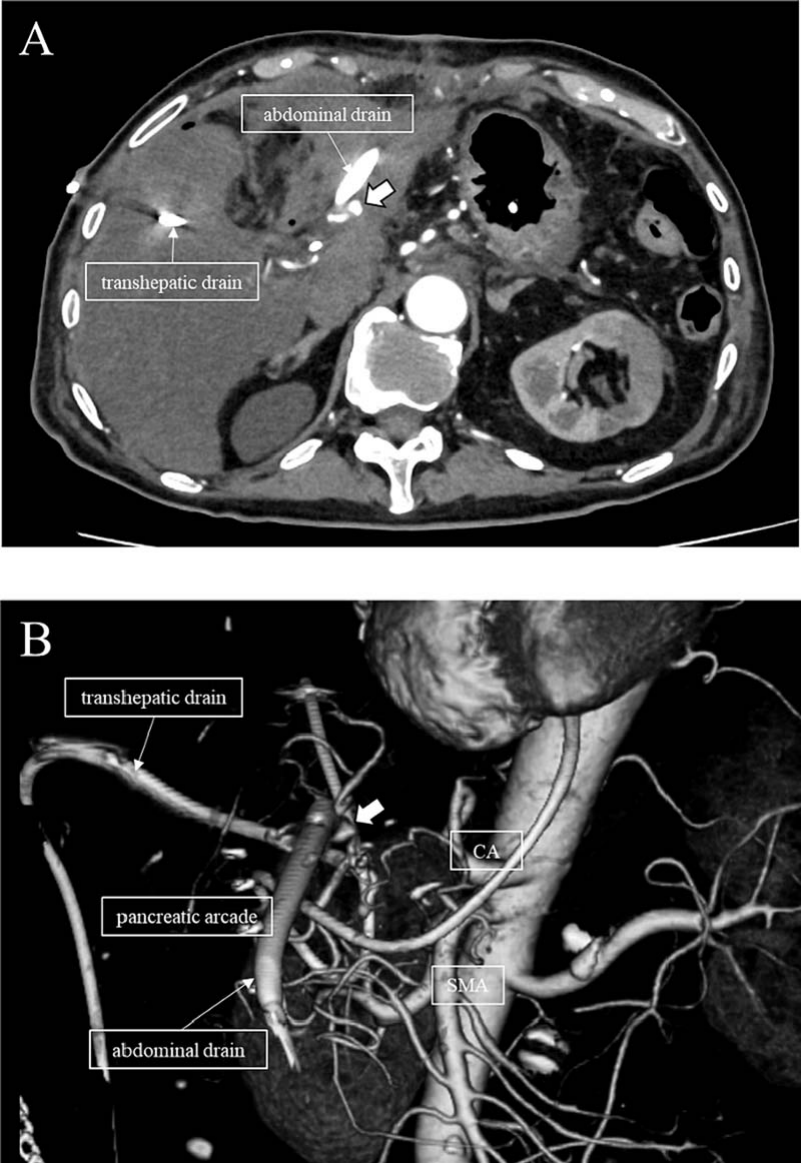
(A, B) CT on postoperative Day 56. Contrast-enhanced CT shows a pseudoaneurysm at junction of the GDA and PHA (arrow).

**Figure 4:**
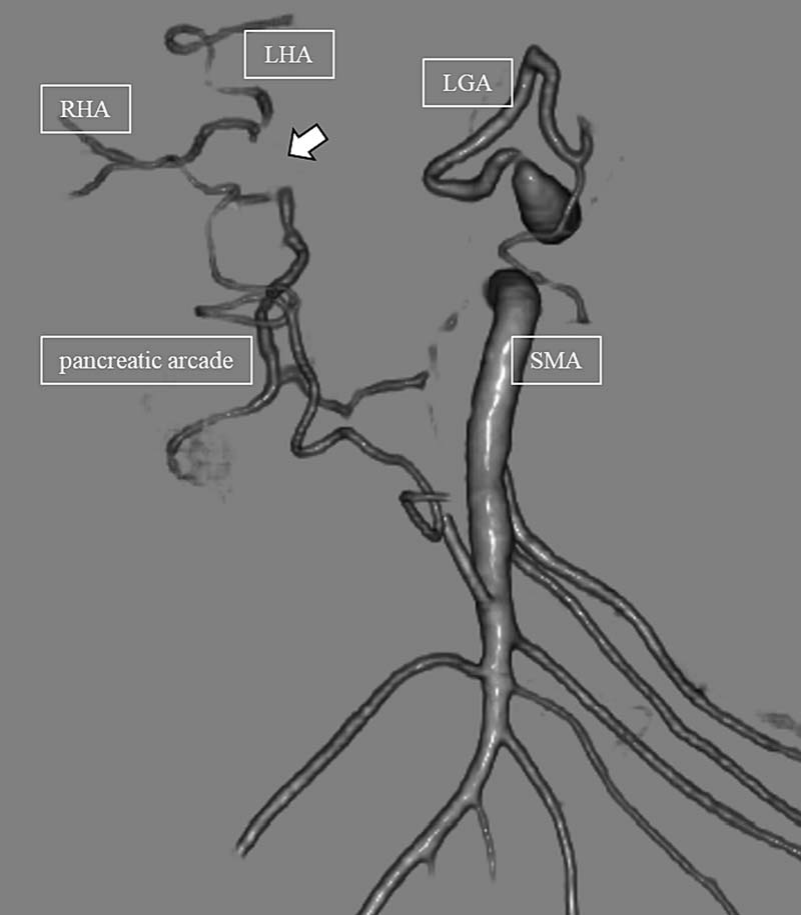
CT on postoperative Day 61. Although blood flow in the PHA could not be confirmed (arrow), the right and left hepatic arteries are preserved.

Subsequently, interventional radiology (IVR) was performed. However, because the inferior pancreaticoduodenal artery (IPDA) was very narrow, the microcatheter could not be followed, and hemostasis with IVR was difficult. Therefore, on POD 48, relaparotomy pseudoaneurysm repair was performed. The abdomen was opened through an inverted L-shaped incision, and adhesiolysis was performed, after which a pseudoaneurysm was revealed at the junction of the GDA and PHA. Four transfixing sutures with 3–0 prolene were used. After confirming that the liver blood flow was maintained, surgery was completed. However, bloody drainage from the drain was observed on POD 56. CeCT revealed a pseudoaneurysm (Fig. 3A and B). IVR was considered difficult because of the narrow blood vessels; therefore, the patient underwent a second laparotomy for hemostasis. A pseudoaneurysm was further observed at the junction between the GDA and PHA. In this operation, we used deeper and wider penetrating sutures than in the previous surgery to completely halt blood flow in the GDA. We confirmed that the blood flow to the liver was maintained; however, it was deemed weak. The patient had an uneventful postoperative course without bleeding, and CeCT performed on POD 61 confirmed the disappearance of the pseudoaneurysm. Although the blood flow in the PHA could not be confirmed, the right and left hepatic arteries were preserved (Fig. 4). On POD 76, CT revealed no liver abscesses or infarctions. The drain was removed on POD 78, and the patient was finally discharged on POD 107 without rebleeding or liver failure.

## Discussion

Pancreatic cancer with celiac artery invasion is often considered unresectable, with a median survival of 6–11 months. DP-CAR has been reported to be a beneficial option for pancreatic body-tail cancer with invasion into the celiac artery, with a median survival of 18 months when combined with preoperative chemotherapy [2]. However, it is considered a very invasive procedure, with one retrospective cohort study of patients who underwent DP-CAR reporting a 30-day mortality rate of 10.3% and a 90-day mortality rate of 16.4%. The most common causes of death were post-pancreatectomy hemorrhage (18%) and organ ischemia of the stomach/liver (45.4%) [1]. Regarding hepatic blood flow, some institutions perform preoperative transcatheter arterial embolization of the CHA to promote collateral circulation and prevent postoperative hepatic ischemia [2, 3]. However, there is no firm evidence to support this. Therefore, this procedure was not performed in this case. We resected the CHA after confirming that hepatic blood flow was not disrupted by clamping.

Hemostasis is achieved through selective microcoil embolization or stent grafting in most pseudoaneurysms that develop after PD. In addition, endovascular treatment has been suggested as the first choice of treatment for bleeding in patients with pseudoaneurysms [4]. Several reports on hemostasis by endovascular treatment of pseudoaneurysms after DP-CAR have been published [5, 6]. Although we also attempted to stop the bleeding initially with endovascular treatment, this procedure was interrupted because the microguidewire could be inserted into the IPDA, the microcatheter could not follow, and surgical hemostasis was achieved.

Takahashi *et al.* [7] previously reported that ligation of the aneurysm and resection of the GDA were performed for a pseudoaneurysm found in the CHA stump on POD 32 after DP-CAR. In this case, there was no description of the vascular run. There have been several reports of successful DP-CAR with concomitant resection of the GDA; however, all were in patients with vascular mutations, such as in the left collateral hepatic artery or right collateral hepatic artery, and none were reported in patients without vascular mutations [8, 9].

In this case, relaparotomy repair was performed for a pseudoaneurysm at the junction of the GDA and PHA. Preoperative CT showed no vascular variations. CeCT on POD 56 confirmed blood flow in the left and right hepatic arteries but not in the PHA. The CHA provides the main blood supply to the liver; however, the right and left accessory hepatic arteries and the inferior phrenic artery also play a role. Therefore, recent studies suggest that the CHA is not essential for parenchymal survival [10]. Collateral vessels are identified 4 h after vascular ligation and increase in size and number over the next 6 months [11]. Therefore, we speculate that collateral blood channels from the inferior phrenic artery and vessels of the lesser diaphragm had developed and hepatic blood flow was preserved in our case. In a search of the literature, we did not find any cases in which hemostasis was performed for a pseudoaneurysm at the junction of the GDA and PHA and hepatic blood flow was maintained by collateral blood vessels in a patient without a vascular anomaly, as in the present case. This suggests that IVR remains the best technique to achieve hemostasis for pseudoaneurysms, but, even when hemostasis by IVR is difficult, following some postoperative days, collateral vessels develop, and hepatic blood flow may be maintained even after relaparotomy pseudoaneurysm repair. Re-relaparotomy pseudoaneurysm repair after DP-CAR may be useful after some postoperative.
